# Adiponectin serenades ceramidase to improve metabolism

**DOI:** 10.1016/j.molmet.2017.01.011

**Published:** 2017-02-01

**Authors:** Saskia Reibe-Pal, Mark A. Febbraio

**Affiliations:** Cellular & Molecular Metabolism Laboratory, Diabetes & Metabolism Division, Garvan Institute of Medical Research, 384 Victoria Street, Darlinghurst, NSW, 2010, Australia

Adiponectin and its signaling through the receptors AdipoR1 and AdipoR2 rank highly in the ongoing search for the holy grail that might improve insulin sensitivity, modulating inflammatory responses, and regulating energy metabolism in patients with obesity and/or Type 2 Diabetes (T2D). Adiponectin is secreted by adipose tissue and binds to its ubiquitously expressed receptors AdipoR1 and AdiopoR2. Over-expression of adiponectin promotes insulin action and improves insulin resistance as well as suppressing inflammatory responses. Over 15 years ago, Philipp Scherer and colleagues demonstrated that intraperitoneal injection of full-length adiponectin into mice reduced plasma glucose levels and suppressed gluconeogenesis, revealing the liver as a major target tissue of adiponectin [1]. Since then, many studies have tried to delineate the molecular mechanisms and signal transduction pathways behind this seemingly wonder adipokine, with a view to the development of novel therapeutics to treat T2D.

In this issue of *Molecular Metabolism*, Holland and colleagues studied transgenic mouse models that over-express AdipoR1 and AdipoR2 in adipose tissue and liver to assess the impact of AdipoR signaling on glucose and lipid metabolism [2]. This is the first time the over-expression of the receptors could be controlled by using an inducible tetracycline element, which allowed the pathway to be switched on following full maturation. Holland et al.'s work focuses on the effects of enhanced ceramidase activity as a result of enhanced adiponectin signaling, supporting their previous work [3]. In the present study, they demonstrate that over-expression of AdiopR1 and AdipoR2 improves whole body glucose metabolism and hepatic insulin sensitivity and opposes hepatic steatosis in obesity.

In their previous work, Holland and colleagues demonstrated that ceramide metabolism predominantly influenced the insulin-sensitizing effects of adiponectin on its primary target tissue, the liver [3]. In this recent publication, the authors extended this work by showing that over-expressing AdipoR1 and AdiopoR2 not only in liver but also in adipose tissue increased ceramidase activity in mesenteric and gonadal fat pads and liver lysates, respectively. When mice were challenged with a HFD for 8 weeks, switching on the over-expression of AdiopR1 and AdipoR2 in adipose tissue or liver resulted in improved glucose tolerance during OGTTs compared with littermate control mice (WT). The AdiopR1 and AdipoR2 mice also showed increased whole body insulin sensitivity. These findings were confirmed by hyperinsulinemic-euglycemic clamp studies.

In an attempt to decipher the molecular mechanisms that would link increased adiponectin-receptor-signaling with improved glucose homeostasis, the key fuel sensing kinase AMP-activated protein kinase (AMPK) appeared to be a logical target. Indeed, it was shown more than a decade ago that binding of adiponectin to its receptors AdipoR1 and AdipoR2 results in the activation of AMPK, which stimulates glucose utilization, suppressing hepatic gluconeogenesis and de novo lipogenesis, and promotes fatty acid oxidation [4].

Holland and colleagues have previously shown, however, that the activation of ceramidase downstream of adiponectin binding to its receptors is independent of the activation of AMPK [3]. Acid ceramidase hydrolyzes ceramides to sphingosine and free fatty acids. When sphingosine is phosphorylated by sphingosine kinase, sphingosine 1-phosphate (S1P) is produced which then acts as signaling molecule itself. Indeed, other groups have shown that S1P overexpression prevents ceramide accumulation and ameliorates muscle insulin resistance in high-fat diet-fed mice [5]. Overexpression of acid ceramidase upstream of S1P appears to have the same effect within adipose tissue in preventing hepatic steatosis and systemic insulin resistance (Figure 1) [6].

Why is hydrolysis of ceramides beneficial when regulating insulin sensitivity? Ceramides are an important key factor in understanding regulation of weight gain and glucose intolerance as they increase during obesity and promote insulin resistance. Ceramides are synthesized by six ceramide synthase enzymes (CerS). Overexpression of CerS6, for example, correlates with insulin resistance, and CerS6-deficient (CerS6D/D) mice exhibit reduced C16:0 ceramides and are protected from high-fat-diet-induced obesity and glucose intolerance [7].

However, a key question remains: Is the activation of AMPK upon adiponectin signaling dependent on S1P being produced or will AMPK also be activated independently of ceramidase activation? If both act independently, and both events result in insulin-sensitizing, anti-inflammatory and anti-apoptotic actions, what is the reason for this parallel signaling? Holland and colleagues present further in this issue that in WT mice, injection of AdipoRon, an adiponectin receptor agonist, resulted in a severe increase in ceramidase activity from liver lysates, indicating that adiponectin agonism may be a beneficial therapeutic for the treatment of hyperglycemia and function via a ceramide-lowering mechanism.

Can the positive effects of adiponectin binding to its receptors be used in treating obese and insulin resistant patients as well as patients with obesity induced non-alcoholic fatty liver disease (NAFLD) or the more severe non-alcoholic steatohepatitis (NASH)? One challenge that remains is that the Kd for small molecules like AdipoRon binding to AdipoR1 and AdipoR2 is much higher than that of the full-length domain of adiponectin [8], so a high-affinity small-molecule activating the adiponectin cascade remains to be identified. Another open question remaining is the effect of constant over-activation of AdipoR1 and AdiopR2 when used as therapeutic intervention. Adiponectin is abundant in the bloodstream so the number of receptors appear to regulate the intensity of downstream activation, as seen by the results of Holland et al. in this issue [2]. It is possible, therefore, that constant over-activation of the receptors might result in a negative feedback loop that impacts receptor expression levels and, thereby, reduces the number of receptors over time.

While the search for new drugs to treat T2D and fatty liver disease continues, one intervention that also improves insulin resistance and decreases fatty liver is physical exercise. It has been shown that AMPK is markedly up-regulated in skeletal muscle upon exercise [9]. In addition, sphingosine as well as S1P levels increase in the circulation following exercise [10]. Hence, exercise seems to be a therapeutic intervention that taps into the same pathways as over-expression of acid ceramidase or AdipoR1 and AdipoR2 receptors to regulate energy metabolism. This raises the question whether the double action of a receptor agonist plus the implementation of a regular exercise regime might lead to promising results in treating obese and insulin resistant patients as well as conditions like NAFLD or NASH. In conclusion, this recent study by Holland et al. [2] certainly supports further the idea that this pathway is a promising future therapeutic target for treatment of obesity-related metabolic disease.

## Figures and Tables

**Figure 1:**
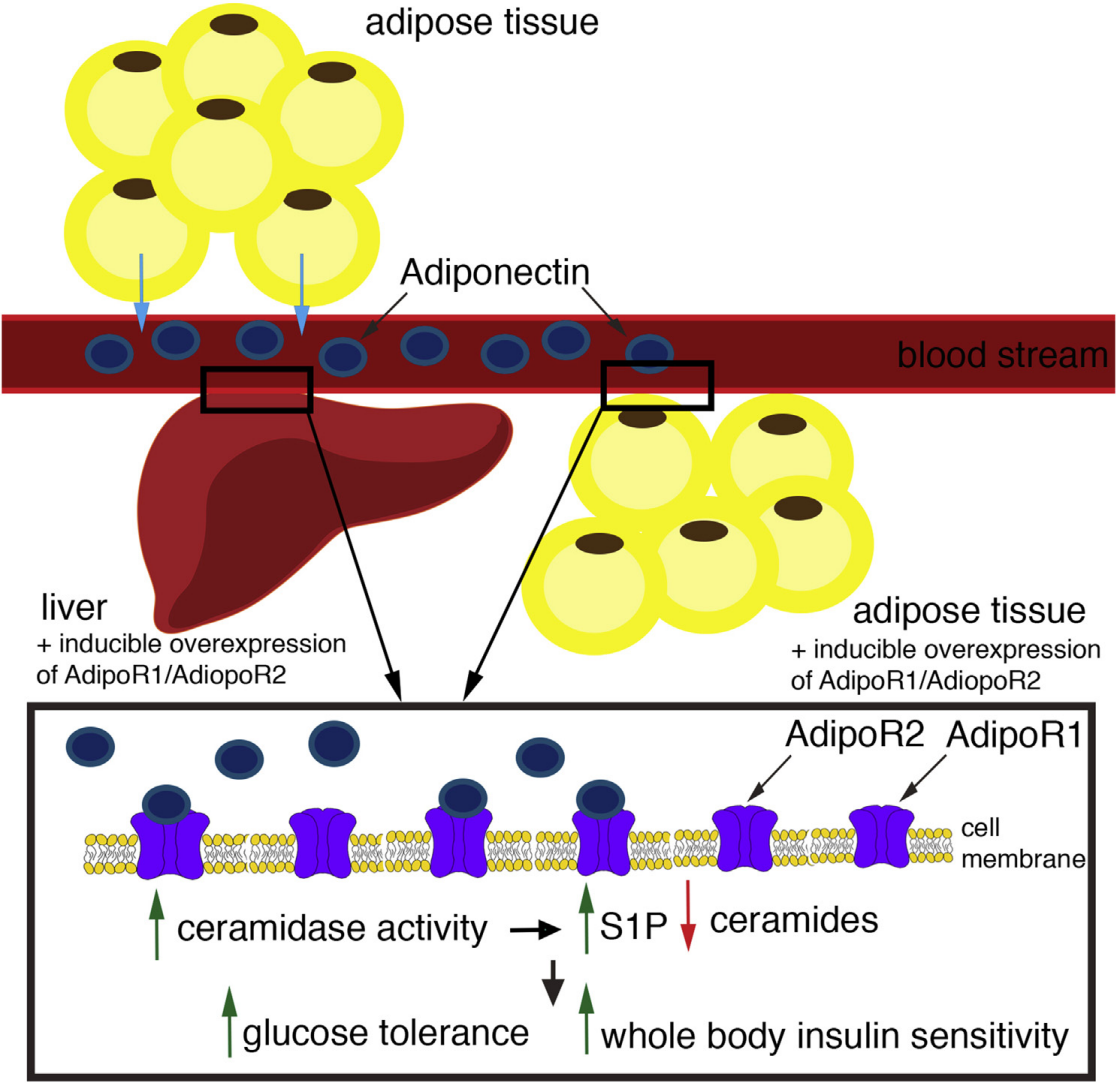
Adiponectin is secreted into the blood stream from adipose tissue. It binds to its receptors AdipoR1 and AdiopoR2 in liver and adipose tissue. Both tissues can be induced to over-express those receptors. Over-expressing AdipoR1 and AdiopR2 leads to an increased number of ligand–receptor interactions which then leads to elevated ceramidase activity. This results in an increase in cellular S1P abundance and a decrease in cellular ceramide abundance. In animals that over-express AdipoR1 and AdiopoR2 glucose tolerance is improved as well as whole body insulin sensitivity.
